# When Doctors and AI Interact: on Human Responsibility for Artificial Risks

**DOI:** 10.1007/s13347-022-00506-6

**Published:** 2022-02-19

**Authors:** Mario Verdicchio, Andrea Perin

**Affiliations:** 1 Department of Management Information and Production Engineering, University of Bergamo, Bergamo, Italy; 2 Berlin Ethics Lab, Technische Universität Berlin, Berlin, Germany; 3 Facultad de Derecho, Universidad Andrés Bello, Santiago de Chile, Chile

**Keywords:** Artificial Intelligence, Responsibility, Moral agency, Due diligence, Principle of confidence, Medicine

## Abstract

A discussion concerning whether to conceive Artificial Intelligence (AI) systems as responsible moral entities, also known as “artificial moral agents” (AMAs), has been going on for some time. In this regard, we argue that the notion of “moral agency” is to be attributed only to humans based on their autonomy and sentience, which AI systems lack. We analyze human responsibility in the presence of AI systems in terms of meaningful control and due diligence and argue against fully automated systems in medicine. With this perspective in mind, we focus on the use of AI-based diagnostic systems and shed light on the complex networks of persons, organizations and artifacts that come to be when AI systems are designed, developed, and used in medicine. We then discuss relational criteria of judgment in support of the attribution of responsibility to humans when adverse events are caused or induced by errors in AI systems.

## Introduction: AI and Responsibility

When humans and Artificial Intelligence (AI) systems interact, there is an issue at stake about who or what can be held “responsible” — and hence possibly “liable” — for adverse events that may derive from this interaction. In this regard, some authors question whether AI systems can or will ever be considered “moral entities” and therefore “responsible entities”, and if so, which conditions are necessary and sufficient for artificial entities to be treated as Artificial Moral Agents (AMAs: Allen et al., 2000; Behdadi & Munthe, 2020).^1^

However, the idea of conceiving AI systems as “moral agents”^2^ and therefore “responsible entities”^3^ has been criticized for at least two reasons.

Firstly, from a conceptual point of view, AI systems are entities to which one cannot ascribe the “moral agency” that is necessary for them to be considered “responsible”. The European Group on Ethics in Science and New Technologies (European Commission, 2018, p. 9–10) has already made a statement along this line: “no smart artefact or system — however advanced and sophisticated — can in and by itself be called “autonomous” in the original ethical sense (…). The ability and willingness to take and attribute moral responsibility is an integral part of the conception of the person on which all our moral, social, and legal institutions are based. Moral responsibility is here construed in the broad sense in which it may refer to several aspects of human agency, e.g., causality, accountability (obligation to provide an account), liability (obligation to compensate damages), reactive attitudes such as praise and blame (appropriateness of a range of moral emotions), and duties associated with social roles. Moral responsibility, in whatever sense, cannot be allocated or shifted to “autonomous” technology”.

Secondly, focusing on practical implications of this debate, many scholars have pointed out that attributing responsibility to an AI system may take responsibility away from the humans who have, in one way or another, contributed to the operations of that AI system (Braun et al., 2020; Bryson, 2010; Johnson & Miller, 2008; Johnson & Verdicchio, 2019; Sharkey, 2017; Tonkens, 2009). In fact, if “AIs are not moral agents, then someone needs to take responsibility for what they do” (Véliz, 2021, p. 3). On the contrary, if AIs are considered moral (as well as legal) agents, then it might be argued that human beings would share responsibility with AI systems, or even that no human being would be responsible for what systems might do and for the harmful events they might cause.

AI systems fit into human relationships in multiple ways: by performing socially useful functions and by introducing risks connected to those functions. Changes in the paradigms adopted in AI technology have expanded the variety of those risks.

The rule-driven, top-down approach of the AI of the beginnings, also known as GOFAI, good old-fashioned AI (Haugeland, 1989), prescribed the encoding of knowledge into a computer in the form of axioms and general laws that mimic deductive reasoning, with the aim of automatising the formulation of a solution to a problem in accordance with the knowledge stored in the computer. The risk here relates to the encoding of knowledge: such knowledge might be incomplete, contradictory, obsolete, or plain wrong, leaving the AI incapable of formulating a proper solution to a problem or, worse, providing a solution that not only does not solve the problem but harms people.

The more recent data-driven, bottom-up approach of Machine Learning (ML) prescribes that patterns, schemes and general laws are searched by computers among a vast amount of data by means of statistical inductive processes. The basic idea underlying ML is to tweak the parameters of complex mathematical functions computing those processes depending on how well their output matches the goals for which the functions were introduced in the first place. Those goals typically consist of tasks of data classification (e.g., distinguishing X-rays of patients with breast cancer from healthy ones), data clustering (e.g., grouping the users of a streaming service in accordance with their tastes), and outlier detection (e.g., notifying a credit card company of an unusual instance of purchase).

The stark difference between ML and GOFAI, however, is not about the goals, but about the way they are achieved: the role played by the programmers of AI technology has radically changed in this paradigm shift. AI programmers are not called to program knowledge and rules into computing machines, but to feed data and tweak complex mathematical functions until they are able to process (i.e., classify, cluster, detect) data in accordance with the programmers’ objectives. In GOFAI, humans set the goals and make the rules to achieve them. In ML, instead, humans still set the goals, but the rules are developed automatically inside the mathematical functions running on the computers in accordance with some general algorithms regulating the alteration of the functions’ parameters. What happens inside an ML system is too complex and quick for human programmers to keep in check. The only aspect programmers can measure is whether the data whose classification was already known to them have been classified correctly by the system as well. If this happens for most known data at the programmers’ disposal, the ML system is said to have “learned” the task, and it is considered ready to be applied to new data to be processed. There is no complete or proven theory on how such results are achieved. Therefore, ML systems are called “black boxes”: humans can only see what goes in (the input data) and what comes out (the classification in output), but not what happens in-between. This paradigm shift in AI comes with a drastic decrease in the direct involvement of programmers in the creation of computational systems that may be used to automatise what has traditionally been performed by humans.

The risks of harmful outcomes are thus increased: in addition to the possibly ill-encoded knowledge of GOFAI, the rather mysterious knowledge created in a black box of ML needs to be harnessed. This task has significant implications on responsibility in AI, which this work is aimed at analyzing.

In the first part, we take a stance against the recognition of a “moral personality” in AI systems or the attribution of a “moral agency” to them. We will show that the principle of responsibility, as a cardinal rule of human interactions, is applicable to “moral entities” endowed with deliberative autonomy (Sect. 2) and self-awareness about said deliberative autonomy (Sect. 3). AI systems, even if hypothetically provided with a high degree of autonomy, are devoid of self-awareness and are therefore not moral entities. Hence, it is up to humans, as moral entities, to create technological conditions that make it possible to manage these risks and prevent the adverse effects that may arise. Those humans are to be considered then responsible if something goes wrong (Sect. 4). We will then evaluate the reasons and the implications of this human-AI relational philosophy by considering the use of AI diagnostic systems in medicine (Sect. 5) and draw our conclusions and propose guidelines for future work (Sect. 6).

## Intentional Agency and Compatibilist Accounts of Human Moral Responsibility

In this Section, we will outline a compatibilist account of individual responsibility. This approach will allow us to argue that the notion of “moral agency” can be attributed only to humans, and therefore it is not applicable to artifacts.

The idea and the principle of “individual responsibility” are traditionally based on the conception of a human being as a “moral entity”. In turn, the notion of “moral agency” is linked to the premise of considering *intentional* action as typically *human*. In fact, philosophers dealing with the question of responsibility have founded their peculiar reconstructions of their imputation theories based on conceptual macro-containers variously called “will”, “voluntariness”, “spontaneity”, “freedom”.^4^ This is why an outcome of a human’s behaviour is not imputable to their responsibility — as their own fact — if it does not depend on their “cognitive-volitional powers” (Civello, 2017, p. 312).

So, a person is responsible in that they are presumed to be capable of acting according to their own “free will”. This allows us to state that the agent could have acted differently; that they had capacity and the possibility to act otherwise. The responsible agent is by definition “free” or “able” to choose whether to comply with or breach a certain rule of conduct. That is the reason why they must respond for the breach of that rule.

Nevertheless, a question arises about whether “moral autonomy” meant as “possibility to act otherwise” is compatible with the impossibility of the “ultimate moral responsibility” (Strawson, 1994). The notion of *ultimate* moral responsibility refers to the idea that human beings should be considered as “uncaused causes”, that is, the idea of free will, where “free” means “uncaused”. However, the possibility of such an idea has been convincingly rejected by several philosophers.^5^ According to the “free will skepticism”, “what we do and the way we are is ultimately the result of factors beyond our control and because of this we are never morally responsible for our actions” in the *ultimate* sense explained above (Caruso, 2019, p. 43).

The reconciliation between *responsibility*, *moral agency* and the *impossibility of ultimate moral responsibility* is possible within the *compatibilist* conception of moral responsibility. According to such conception, “the fact that factors outside a person’s control influence his or her actions is *compatible* with the idea that *the person* is the ultimate causer of his or her actions. Thus, the person is culpable (i.e., responsible) for the action that he or she causes, even if external factors contribute to the production of such actions” (Coppola, 2021, p. 14; see Caruso, 2012 also for criticism).^6^ Moreover, human beings are responsible precisely because they are deeply conditioned by factors beyond their control. Otherwise, those prevention strategies adopted by contemporary legal systems — based both on the threat of a sanction in case of a law breach (negative general prevention) and the communicative or motivating meaning of its application in society (positive general prevention) — would not make sense. If our decisions were not influenced by threats and encouragements, prevention would not be effective.

Within a compatibilist conception of the principle of “responsibility”, the notion of “human agency” sets aside the classical (and impossible) idea of “freedom” intended as absence of causes. This notion rather reflects a hypothetical (i.e., presumed) space of “deliberative normality”. One could say that a “free” (=responsible) agent is “normally conditioned” in their will, rational deliberation, and action.^7^ In other words: we are not free, but sometimes it is as if we were^8^; we cannot act otherwise, but sometimes we judge our conducts as if we could.

In this way, we can state that the concept of responsibility also “includes a specific psycho-motivational constitution of the responsible subject: we think she or he is answerable in the sense of being an autonomous person, able to take on her or his responsibility, and equipped with capabilities such as judgement and the faculty of reflection” (Loh, 2019, p. 2).

Borrowing from the terminology of philosophy of law, this can also be expressed in the following terms: the “responsible” agent is the one suitable to be “motivated” by moral and/or legal rules of conduct and, therefore, they are a possible recipient of sanctions. Hence, when a breach of a certain rule of conduct occurs, the imputation of responsibility is justified if we assume (unless proven otherwise) that the agent has “normal” subjective skills^9^ and that they were acting in “normal” (not exceptional) contextual or concurrent circumstances.^10^

So, the question at stake is the following: if “free will” means that “nothing external to the party in question was forcing its actions and that it did what it “intended” to do”, why couldn’t this concept apply «almost equally to humans, many animals, and autonomous robots”? From this perspective, a robot appears to be “just as blameworthy as a human for its actions and just as deserving of consequences for its actions” (Mulligan, 2018, p. 592). In other words: if a human — just as an artifact — is subject to the principle of causality,^11^ why would only the former and not also the latter be responsible?

## Autonomy and Sentience in Human Moral Agency

Floridi & Sanders, (2004) argue that artificial autonomous agents can be considered moral agents, and that they constitute an instance of “mind-less morality”. In disagreement with them, Véliz, (2021) argues that “the main reason why algorithms can be neither autonomous nor accountable is that they lack sentience”. Sentience “is necessary for moral agency” because it implies the necessary moral understanding to be morally responsible.^12^ In what follows, we argue for the latter stance.

The requirement of deliberative “freedom” (understood as deliberative normality or responsible caused-will: as we illustrated in Sect. 2) is not sufficient for the purposes of the attribution of moral agency. This sort of autonomy must be accompanied by a second requirement: the self-awareness of the capacity to decide, the ability to understand it as “one’s own”, and therefore also to take the resulting responsibilities.

This second requirement, which we could also define as “feeling of freedom”, characterizes humans and makes them responsible on a relational level (towards others with whom they potentially interact). Self-awareness or self-perception of freedom is what allows everyone to assume a fact as “their own” or “of others”. Instead, although hypothetically endowed with a high level of autonomy,^13^ the artifact is devoid of consciousness, it does not perceive nor could it assume the fact as “its own”, either individually or socially (that is, on a relational level). The absence of this precondition for “moral agency”, individually and socially perceived as such, prevents the same paradigm of human responsibility from being applied to the artifact.

The need of this self-awareness to be morally responsible derives from the objectives that we assign to the principle of responsibility. A sort of retribution still underlies contemporary utilitarian concepts of punishment. In fact, imposing sanctions can be useful for *motivating* lawful behaviors only if society attributes a symbolic meaning to individual responsibility. This symbolic meaning is essentially what lets us affirm that certain harmful conduct is “blameworthy”. The difference with respect to the purely moral conceptions of retribution consists in overcoming the “prejudice” according to which “guilt and responsibility presuppose a “free will””^14^ (Sect. 2). This makes it possible to abandon vengeful impulses^15^ and to attribute “secularly” communicative, remedial, or strictly restorative purposes to the attribution of responsibility and the application of sanctions.^16^

So, instead of being a question of revenge or pure retribution, the principle of individual responsibility should lie in the meaning and the preventive effect that its imputation to someone for their actions may assume in society. That is why this attribution requires that both the agent and society could assume the fact as their own. We need to recognize that the agent who is held responsible for their conduct can assume the responsibility for what they did.

If deliberative normality (autonomy)+self-consciousness (sentience)=human moral agency (*responsible* although *caused* will), then said “moral agency” is not applicable to an AI system. In the face of an adverse event caused by an AI system, there is no reason that justifies an attribution of responsibility to the artifact. The imputation would have not any communicative meaning towards society. No one would assume the fact as “the AI system’s own”. No one would feel that responsibility for what happened falls on it.

We now focus on the practical reasons underlying our standing. In addition to their lack of moral agency, we will consider why artificial systems, even though endowed with a high level of autonomy, should not act autonomously, nor shall they share responsibilities with humans.

## Human Responsibility Around “High-risk” AI Systems

### Towards a Preventive System

The previous considerations do not entail that an artifact cannot be made the recipient of consequences for its “actions”. However, the reason behind the application of certain consequences should only be the “special negative prevention”. This means that the goal of any normative system should be to provide and take measures so that the artifact does not cause more harmful events.

AI systems are meant to fit into society by enabling humans to carry out socially useful activities with greater speed and lower costs. However, they also generate serious risks that are classifiable in terms of severity and social harm. That is why it is up to humans, as moral entities, to create technological as well as normative conditions that make it possible to take advantage of automation, while managing those risks and preventing the unacceptable adverse effects that may arise. In other words, we should treat artifacts for what they are: both *useful instruments* and *dangerous agents lacking morality*.

In the history of European modern thought about the principle of “responsibility”, as well as in contemporary legal systems, we already find models that consider certain agents not morally imputable, but socially dangerous or characterized by a certain “social risk”. Various versions of 19^th^-century positivist criminology proposed systems of “responsibility” based on the idea that, “scientifically, there are only causes, not faults”.^17^ Therefore, the type of punishment was conceived based on the “type of perpetrator”, that is, we could say, the expected or foreseeable “type of criminal risk”, and the legal consequence was therefore pure prevention: resocialization, neutralization, or mere intimidation. What mattered was “to seek the causes of the crime: to prevent, not to blame”. According to the culture of 19^th^-century philosophical positivism, “science could really guide morality”.^18^

Today, this paradigm is partially welcomed by legal systems that provide post-trial security measures that can be applied to non-responsible and socially dangerous offenders.^19^ Of course, the fundamental difference from back then is the need to respect human rights.^20^ Moreover, the non-responsibility depends on an “incapacitation” (e.g., because of a major mental illness or serious personality disorders) that allows to rule out moral agency (= autonomy + sentience).

*Mutatis mutandis*, an analogous idea (anti-retribution, utilitarian, purely preventive) should underlie any normative system that intends to regulate the relationships between human beings as moral entities, and AI systems as non-moral sources of risk. Artifacts should be conceived as “centers of indictment” in the same way as potentially dangerous agents (“caused causes” without moral agency). The consequences associated with certain adverse events possibly caused by AI systems may befall them (e.g., in terms of neutralization), but the duties and responsibilities relating to such events must befall the humans who determined or otherwise conditioned the operation of the AI systems (e.g., programmers, users, etc.).

Such idea would be about setting up a system of “preventive law”, the activation of which would occur under two fundamental conditions: (1) a verification that an AI system has caused, or is about to cause, or could cause damages worthy of intervention; (2) a judgment of “objective risk”. The reaction would be preventive: e.g., suspension, reprogramming, or even destruction and prohibition of use.

This is indeed, as we will see in what follows, the (human-AI) relational philosophy underlying the new *Proposal for a regulation of the European Parliament and the Council laying down harmonised rules on Artificial Intelligence (Artificial Intelligence Act) and amending certain Union legislative acts* (EU Artificial Intelligence Act, henceforth AIA-21).^21^ In fact, the proposal excludes the possibility of assigning to AI systems any status as a legal person, with rights and duties. By adopting the so-called “risk-based approach”, the AIA-21 classifies AI systems based on the level of risk: (i) unacceptable, (ii) high (e.g., medical devices^22^), (iii) low or minimal, and it applies risk assessment standards according to the risk category attributed to an AI system. The responsibility to fulfill the relevant obligations and standards rests entirely with humans who design, manufacture, market, and use the AI system (Floridi, 2021, p. 219).

### Meaningful Human Control and Due Diligence

What has been observed so far reflects a principle already upheld in the literature and progressively confirmed also on a regulatory level: “technology ought to be designed in such a way that responsibility distribution remains “tethered to humans”” (Braun et al., 2020; Johnson & Miller, 2008; Romeo Casabona, 2020). This need for “tethering” can be met by a standard called “meaningful human control”. “This means that humans — and not computers and their algorithms — should maintain ultimate control, thus being able to be morally and perhaps legally responsible for it as well” (Romeo Casabona, 2020, p. 183).^23^

The General Data Protection Regulation (henceforth GDPR-16) has already adopted a similar approach. In fact, according to art. 22, decisions in the context of AI systems cannot be fully automated, i.e., based “solely on automated processing”.^24^ As it has been argued, this could be interpreted as to establish a “right to information and explanation” (Selbst & Powles, 2017; Watson et al., 2019), and therefore entail that “black box” systems, which don’t allow any “meaningful human control”, nor any explanation, should be prohibited (as we will argue in Sect. 5).

Dealing with the “right to an explanation” and the limits of full automation by recent regulation is of critical importance because, although there are still few cases in which AI systems take decisions autonomously, there is an assumption that “AI systems will eventually outperform human practitioners in terms of speed, accuracy, and reliability”.^25^

In this regard, the claim that opaque AI systems must be prohibited based on the GDPR-16 could be controversial (not so under the AIA-21, as we shall see below).^26^ One could sustain that the GDPR-16 only establishes the data subject’s right not to face the consequences of the data processing alone, even though an explicit consent to an automated decision is given, thereby requiring that someone is responsible for the automated decision. However, “black boxes” don’t allow for human interpretation and explanation,^27^ thereby conflicting with the core idea of patient-centered medicine (Bjerring & Busch, 2021, p. 358, 360), that is, the bioethical principle of autonomy and the necessity of the informed consent (which is required by Arts. 22.4 and 9 to except the general prohibition).

We then claim that the “meaningful human control” standard can make it possible to comply with those general provisions. In fact, it can allow for the assignment of specific duties of assessment of the objective risks of artifacts and of control during their use, based on schemes of *compliance* and *due diligence* established for designers, manufacturers, distributors, and end users. These schemes have a preventive aim and imply the existence of organizational and risk management structures and procedures aimed at preventing not only adverse events but also decisions that are consistent with the conclusions of an AI system but are not validated by human experts (Romeo Casabona, 2020).

Conveniently, similar schemes are now adopted by the above-mentioned AIA-21. In fact, it establishes imputation centers and relevant duties: design conditions that allow for subsequent evaluations and controls^28^; certifications of conformity; risk assessment duties (compliance and risk assessment, functional to the fulfillment of preventive duties, such as the provider’s duty of information^29^); duties of surveillance, control, and notification of adverse events (post-release)^30^; duties of care when using or otherwise interacting with AI systems.

In what follows we focus on these “duties of care” during the use of artifacts and their relationship with the “risk assessment duties” and *ex ante* risk communication, with a specific focus on the medical sector.^31^

## Case Study: AI and Medical Malpractice

Let us consider the following case study: a medical doctor who uses a diagnostic imaging system makes a “false negative” diagnosis based on the output of the system, which fails to indicate the presence of cancerous tissues.

In such a situation, two general questions arise (Perin, 2019): (i) Is there a desirable level of automation in medical care and, more specifically, is full automation desirable? (ii) How should a medical doctor behave if they do not agree with the diagnosis proposed by an AI system?

In addressing the first question, in addition to our general considerations about moral agency, responsibility, and meaningful human control, we will now consider four arguments against full automation in the context of healthcare. As we have already pointed out, this is of critical importance in the light of the statement that AI systems will eventually outperform human practitioners in terms of speed, accuracy, and reliability. After explaining why total automation in medicine is not desirable — mainly on bioethical grounds, we will then focus on the second question concerning the imputation of responsibility.

### Four Arguments Against “Full Automation” in Medicine

**(1)** The first argument against full automation in medicine stems from the *learning* techniques that characterize some AI systems. The information and data given to the systems originates from the work of humans in terms of collection, classification, and categorization and, therefore, it cannot be 100% reliable and neither can the performance of the machine’s learning model.^32^ Still, this argument is not conclusive. Preliminary reports on the reliability of AI systems seem to show that the *accuracy* of certain systems is already higher than that of trained professionals; thus, although we should take those reports «with a grain of salt» (Bjerring & Busch, 2021, p. 345), it would be questionable to claim that AI systems are less reliable than professionals (see, for a systematic review on diagnostic inaccuracies, medical biases and resulting errors, Saposnik et al., 2016). Coherently, the accuracy standards required to authorize the use of AI models are progressively higher.^33^

**(2)** The second argument refers to the levels of *robustness* which AI systems can ensure: as it has been demonstrated by cases reported in literature (Mirsky et al., 2019), AI systems suffer from *vulnerability* and might be manipulated by malware. The data used by the systems and determining their decisions may be stolen, altered, or destroyed, putting their confidentiality at risk.^34^ There are also much more subtle attacks, which do not involve theft, but images manipulated in an invisible way to humans (a few pixels here and there) which, however, overturn the result of the classification by the AI system (Jain, 2019).

**(3)** The third argument involves the *lack of transparency* (i.e., *opacity*).^35^ There are models whose decisions are relatively interpretable, others whose level of opacity is extreme. The interpretation and understanding of the criteria or parameters on which the most advanced “deep” learning systems base their decisions — for example, those that use *convolutional neural networks* — is extremely complex, also for those who developed the original algorithm (Watson et al., 2019).

So, concerning the possible use of those “black boxes” as assistant medical devices, the question would be the following: «If doctors do not understand why the algorithm made a diagnosis, then why should patients trust the recommended course of treatment? Moreover: how to guarantee the exercise of the *right to receive an explanation* established by art. 22 of the GDPR-16? (Selbst & Powles, 2017; Watson et al., 2019)^36^ This provision might entail a general prohibition, addressed to the person responsible for the treatment, to make automated decisions that do not have a “significant human intervention”, i.e., without medical supervision.

As we already noted, the claim that opaque AI systems must be prohibited based on the GDPR-16 is arguable (Sect. 4.2). However, a stricter standard has been more recently put forward by the AIA-21, whose Art. 13 expressly requires a level of transparency that allows for the “interpretability of the model”.^37^

We can thus affirm that there is a clear coherence between the *principle of transparency*, the *right to explanation*, and the *right to consent*, generally recognized in biomedical matters. For this reason, the healthcare professional cannot assume, accept, and apply “blindly” a diagnosis suggested and delivered by an AI system: he or she must understand how and why a certain decision is made, and know how to explain how the system reached a certain decision. This conclusion is thus incompatible with the idea of replacing a doctor with an AI system.

**(4)** Finally, against full automation it has been argued that “Medicine in part still remains an art which can never be fully quantified or solved. There will always be an outlier, always be a niche case, always be confounding factors. And for that reason alone, we will always need some form of human oversight” (Harvey, 2018).^38^ Medicine would then require *intuition* and *creativity* as typically humans’ skills. So, the question at stake is whether only humans can do inferential reasoning which allows establishing new correlations and finding surprising solutions, that is, making “extraordinary abductions” (Aliseda, 2000; Tuzet, 2006). Of course, determining causal correlations and finding explanations is precisely the problem of (medical) diagnosis. Furthermore, we could wonder whether AI systems can make equally unexpected predictions and take innovative actions (like the one which allowed the first COVID-19 diagnoses in Northern Italy in early 2020, thanks to the decision to breach current medical guidelines).^39^ One might respond positively by pointing out at the “creativity” demonstrated by certain AI systems, such as AlphaGo. AlphaGo is a system developed by Google DeepMind and based on “reinforcement learning”, which in 2016 managed to beat the world champion of Go, a traditional Chinese board game. This AI system made an unexpected move during one of the games, which — as it has been argued — would have demonstrated its ability to be “creative”, that is, its ability to act not only based on patterns deduced from experience and learning, but also on completely new rules and strategies.

Such a conclusion relies on a *certain way* of understanding the idea of “creativity”, meant as a capacity to “discover something which wasn’t known before”, to act in an “unexpected” or inexperienced way, “outside of our norms”, from the perspective of common sense and common experience.^40^ Certainly, this meaning of “creativity” could be debated further,^41^ considering the fundamental difference between “understanding” (typical of humans) and mere “predictability” (as attributed to AI systems, which lack “hermeneutic autonomy”^42^). However, such “artificial creativity”, meant as a capacity to obtain information that apparently cannot be derived directly from the data, already existed before AlphaGo. Indeed, it must be recognized that the treatment of data through machine learning “allows establishing relationships between very diverse data and obtaining conclusions that would be impossible or very difficult to extract from reduced or more homogeneous amounts of data”; furthermore, “big data involves not only a quantitative alteration in the processing of data, of huge amounts of data, but above all a qualitative change, as its treatment makes it possible to obtain information that is apparently not implicit in the data or it cannot be derived or deduced directly from them” (Romeo Casabona & Lazcoz Moratinos, 2020, p. 68).

Perhaps the real novelty was rather the context and the level of complexity of the unpredictable and unexpected performance of the system.^43^ Therefore, if the “surprise” for the event that no one could anticipate stems from our limited ability to process data compared to that of the system’s, it appears to be more «trivial» than related to genuine creativity (Delacroix, 2021).

In short: although it could be stated that (1) certain AI systems could be already (at least) as accurate as humans and that risks of errors (error rates) are decreasing, and (4) that the AI systems are somehow “surprising” (or even “creative”), there is still (2) a security risk and (3) the principle of autonomy that applies in biomedical matters implies the patient’s right to receive an explanation and this requires an active as well as responsible participation of the medical doctor in every decision-making process, in which she could still be assisted by AI systems.

Arguable concerns involve therefore bioethical issues stemming from *opacity* and moral as well as legal issues concerning *responsibility*, more than *accuracy* or *efficiency*. This opacity is higher than in traditional evidence-based medicine (EBM)^44^ and of a different kind. Undoubtedly, also practitioners who follow medical guidelines and/or emerging research evidence are often uncertain or radically ignorant about theories on why certain interventions work, or about the reasons behind certain predictions or the risks associated to certain treatments or their benefits (London, 2019, p. 17–19). However, “in ordinary clinical contexts, the clinician guides the patient through the spectrum of available care” (Braun et al., 2020, p. 2) and “ordinary opacity” is more often a question of possession of relevant knowledge or experimental evidence (which is available to a certain degree, although it might be scientifically uncertain, or not yet generally accepted, and often fragmentary and incomplete) and its proper communication to patients.^45^ Instead, when doctors deal with automation, black box AI systems replace human reasoning without providing practitioners and patients with useful causal explanations of the systems’ internal decision procedures between input and output (Braun et al., 2020, p. 3; Bjerring & Busch, 2021, p. 363–364) or about implicit relevant value-laden concepts assumed (Grote & Berens, 2020, p. 209–210). Moreover, those systems make it “almost impossible to assess risks adequately *ex ante* as well as to prove *ex post* which wrongful action has led to specific damage, thus hindering legal evaluation” (Braun et al., 2020, p. 3).

### Doctors’ Responsibility and the Principle of Confidence

With these provisional concerns about full automation in mind, we now focus on the second question: how should a doctor behave with respect to a diagnosis made by an artificial intelligence system?

We need to design a model of distribution and allocation of responsibility consistent with the necessary human medical intervention, but that also allows for the advantages offered by AI systems in medicine, namely, efficiency and cost reduction. A normative model of responsibility consistent with these premises should then generate the conditions for increasing trust in collaborative teams composed by professionals (i.e., humans) and AI systems (Braun et al., 2020, p. 3).

Our proposal in this direction is the so-called “principle of confidence” (Perin, 2019). This principle indicates that a person who takes part in a relational activity in society, can act in confidence that all other participants acted / are acting / will act in compliance with their own duties of care (e.g., when driving a motor vehicle, when working as a team during a surgery). That confidence generates a legitimate or fair expectation, which implies that one has no duty to check constantly that other participants are acting according to their duties of care.

However, like any rule, this one also has exceptions. In fact, if evident and concrete indicators make it possible to foresee that a participant is failing to comply with due care, i.e., they are not acting as it could be expected, then the “legitimate confidence” will be replaced by a duty to act in order to avoid adverse events that may derive from the interaction between the subjects involved in the plurisubjective activity (Kirschbaum, 1980; Mantovani, 1997; Perin, 2020).

In philosophy of law, the principle of confidence reflects the liberal idea that abstractly dangerous activities are allowed until concrete circumstances recommend that a “reasonable and prudent person” refrain from a certain performance, so as not to exceed, in this way, the threshold of the so-called “allowed risk”.

This principle (and, at the same time, imputation criterion) could then be applied, *mutatis mutandis*, to the relationship between a doctor (moral and responsible entity) and an AI system (the non-imputable “third party”) for the construction of normative frameworks aimed to meet the double objective of: (1) protecting the legitimate confidence of the physician in the automated diagnosis, but (2) without justifying or encouraging a tendency to uncritical or blind application of it, recognizing that in qualified cases the doctor has a *duty to contradict* the system’s indication and otherwise she could respond for not having realized that the indication she received was wrong.^46^

Recognizing this limit to the principle of confidence entails asking under what conditions (in each specific case) can a duty be established that imposes taking charge of the error of others. The challenge then consists in determining when, specifically, there could be an objective enforceability of said residual and “relational duty” to face the error of a decision-maker that lies outside the acting person (Perin, 2019; Caputo, 2021, p. 198–199). This normative approach could allow for the determination of a physician’s responsibility in situations like, for example, the following: (1) when the AI model is used beyond the scope for which it was authorized and certified (off-label), so that any expectation would no longer be valid^47^; (2) when the medical doctor trusts an automated indication despite concrete and serious doubts about the reliability of the system.^48^

A possible advantage of an approach based on the “principle of confidence” is that the burden of proof would not shift towards the clinician (as warned by Braun et al., 2020, p. 7–8), because *fair reliance* would be the rule, not the exception, and so would be the exoneration from any responsibility in case of deriving adverse events. Moreover, the maintenance of a residual duty to contradict the system’s indication might discourage “defensive” automatisms (i.e., blind and uncritical acceptance of AI systems decisions) and reduce the loss of the “epistemic authority” of the clinician (discussed by Grote & Berens, 2020, p. 207). In fact, the final decision would never be fully automatic, and the doctor would be accountable, taking the moral – although not always the legal — responsibility for it.

This approach seems therefore to indicate a balanced and flexible solution between fair reliance, a liberal principle suitable for ensuring a reasonable room to maneuver, and due diligence, a deontic basis for holding potential liability for medical malpractice.

Nevertheless, we must recognize that the application of the “principle of confidence” might cause also serious concerns. First, this solution may require being able to affirm that AI systems are normally reliable, but in some cases this conclusion appears somehow premature. Moreover, «in order for the human subject to evaluate the indication of error, he must proceed by checking, examining and evaluating the conclusion or decision-making proposal of the system, otherwise such indications could not be grasped or motivate the agent to make independent decisions» (Romeo Casabona & Lazcoz Moratinos, 2020, p. 83 and following). Secondly, there is a problematic issue affecting the relation between the level of automation of the AI and the human physician. The principle of confidence is generally applied to two or more autonomous and responsible human entities that collaborate or interact in the realization of a specific activity. Here, however, the scenario is somewhat different, because AI systems are not “moral entities” (as we argued in the first part of this paper) and because there are different levels of automation (among which the full automation of healthcare processes, whose admissibility we have already excluded).

### Automation in Medicine and the Principle of Confidence

Bitterman et al., (2020) stated that the “level of AI autonomy is broadly accepted to be crucial to the risk-assessment of AI algorithms, yet its definition and classification is poorly defined”. Therefore, adopting the same scheme of levels of automation applied to autonomous vehicles, they indicate five possible degrees of automation also with respect to the context of AI in medicine: data presentation, clinical decision-support, conditional automation, high automation, and full automation.

This adaptation could enable the application of the principle of confidence in the sense that the more automation increases, the more the confidence increases, the more the responsibility of the doctor decreases and, in turn, the responsibility of the designer and/or the programmer of the AI system increases. Nevertheless, this approach deserves a critical analysis.

This is not the place to shed light on the issues of that proposal in the context of self-driving vehicles, but criticalities are present, perhaps to an even greater extent, in the medical field. It is very difficult to define a concept of “autonomy” of AI systems in medicine, when the algorithms that regulate their operation are strongly dependent on a significant number of human beings who have contributed, directly or indirectly, to the definition of the instructions of such algorithms.

In every computer artifact there is an intrinsic dependence on the action of human beings: those who built the artifact (building hardware) and those who programmed it (writing software). Even in this restricted context, considerations could be made on the automation of the building and programming processes, but since it is a general characterization of Computer Science tools, we want to focus here on the more specific peculiarities of AI systems in the medical field.

In particular, we will focus on two kinds of AI systems: Support Vector Machines (SVM) and Deep Learning (DL) systems, which reprise the distinction between GOFAI and ML, which we made in the first part of this paper. Both SVM and DL are designed to automate classification, that is, the task of analyzing medical data, typically in the form of digital medical images of patients’ organs and classifying them into categories. In the simplest case, the classification distinguishes pathological cases from physiological ones. In SVM systems, quantitative parameters whose values characterize healthy patients in accordance with the expertise of the doctors involved in the design process are explicitly programmed into the system, so that when a digital medical image is analyzed that presents values that fall outside of that “healthy” range, it is classified as pathological and brought to the doctors’ attention. In DL systems, on the other hand, the role of the human expertise is less explicit, because DL systems consist of a neural network (a complex network of simple mathematical functions) that tweaks its own parameters based on a trial-and-error training phase, during which the system is fed with medical images and its classification output is matched against the human doctors’ pre-recorded classification results. When there is a mismatch, the DL system alters its own parameters; when there is a match, the DL system stays the same. This training procedure goes on until the mismatch rate goes below a threshold that is deemed acceptable by the people involved in the design of the system.

Peruzzo et al., (2016) propose the use of an SVM system for the automation of the detection and characterization of malformations in the brain, with particular attention to the corpus callosum. Here is an illustration of the main points of the proposed process, with emphasis on the dependence of this process on human factors external to the AI system.

#### Support Vector Machines for Automated Brain Analysis

Support Vector Machines are classifier systems, i.e., algorithms that group numerical data into sets based on a measurement of the numerical distance between data points: data are considered similar when they are characterized by values whose difference is below a preestablished threshold.

In the proposal by Peruzzo et al., (2016) the numerical data consist of measurements of the dimensions of the corpus callosum of neurology patients. The basic idea is that if these data show a significant difference with the measurements of a physiological corpus callosum, then it is possible that the patient has a pathology and therefore needs further investigations.

The procedure for creating numerical data starting from the patient’s brain involves the following: a magnetic resonance is performed to create digital medical images with MRI technology; these images are processed with standard software (FMRIB Software Library) for the elimination of noise and discontinuity in the image due to intrinsic delays in the resonance machinery; the images are scaled and normalized in relation to the measurements of an “average brain”, such as the one present in the “Brain Atlas” of the Montreal Neurologic Institute; the measurements of the corpus callosum under examination are extracted according to a predetermined geometric model, in terms of area, perimeter, distance between focal points, thickness.

Once the numerical data are created, they are processed by the SVM system, which inserts them into a multidimensional mathematical space, where they constitute a new point, representing the corpus callosum under examination. In the same space there are numerous other points representing the other corpora callosa examined previously, labeled as physiological or pathological based on criteria established by the medical knowledge of the doctors that participated in the classifications.

The SVM system, based on the distance of the new point from the area where the points previously classified as physiological are located, establishes whether it represents a physiological or a pathological corpus callosum. Furthermore, in the latter case, with an operation based on mathematical counterfactuals, the system calculates the displacement in the multidimensional space that the point needs to reach the boundary of the physiological area. This shift, in terms of the parameters involved and the extent of the shift itself, creates an “explanation” that accompanies the classification of the point as pathological. It is far from an explanation in scientific terms, but at least the human expert who receives the result of the classification also gets data to support the decision taken by the AI system. This is a “characterization” of the result that should help the human expert in the “validation” process with further medical investigations.

#### A Case Against Levels of Automation in Medical AI Systems

The complexity of the relationships between prior and distributed knowledge, interdisciplinary and multidisciplinary efforts, numerical modeling and technological implementations that characterize the aforementioned proposal sheds light on the excessive simplification imposed by a framework of levels of automation in AI systems such as the one proposed by Bitterman et al., (2020), where a dichotomy of “clinician” and “AI” is presented, and where “liability” falls either on the “clinician” or the “AI developer”. Whatever the approach that is adopted to ascribe responsibility in the medical field with AI systems, be it meaningful human control, be it the rights to information and explanation, whether one requires ex ante supervision and controls or ex post countermeasures, it is clear that the entities that must be taken into consideration are much more numerous than a single AI system in use in a hospital and the human being who has used it for a particular medical case.

In particular, in the case of the use of SVM systems for the analysis of the corpus callosum, at least the following entities are involved:

- The company that supplied the MRI machinery;
- The company that provided the software for the pre-processing of the images;
- The technicians managing the Brain Atlas, as well as all the hospitals and research centers that provided the data with which the atlas was built;
- The researchers who established the geometric model of the corpus callosum to be used in the experimental study;
- The programmers who created the SVM system used in the experimental study;
- The clinician who uses the results of the SVM system to deal with a case.

We can imagine a series of problematic cases of misdiagnosis (false negative: the patient has a pathological corpus callosum but is not diagnosed as such) in which the responsibility falls on each of the entities listed above:

- The pathology does not appear in the image due to a malfunction of the MRI machinery;
- The pathology does not appear in the processed image due to a malfunction of the image processing software;
- Normalization of the image with respect to the Brain Atlas makes the pathology invisible on the image;
- The pathology alters a characteristic of the corpus callosum that was not taken into consideration by the geometric model established by the researchers;
- The image is classified as physiological due to a malfunction of the SVM system software;
- The clinician considers the SVM system result to be incorrect and decides that the patient’s corpus callosum is physiological.

In this list we have imagined independent events, but of course we cannot exclude cases of accumulation of errors and malfunctions. The intrinsic dependence of AI systems on data and on criteria decided by experts to process such data means that this network of relationships between different human beings and technologies they have developed holds even in a scenario of significant development of AI technology: machinery can always break down or wear out; the models on which the software is based may be incomplete, or become incomplete in case new types of pathology emerge that were unknown at the time of modeling, and so on.

A detailed analysis of this network of relationships is not an exercise that is destined to become obsolete because of technological development. It is indeed a fundamental step for the construction of a system of attribution of responsibility that aims to increase the trust towards these human-AI hybrid systems. Such trust will be based on the speed with which any system malfunction can be identified and the relevant responsibilities correctly attributed.

Due to the explicit description of the characteristics of the analyzed organs, SVM-based techniques fall into an operational category called “feature engineering”. More recent advances in ML, specifically in the field of DL, have focused more on the use of data classified by human experts without any indication on characterizations or quantifications of features. With this shift, medical image classifiers that had taken years to develop could be designed, trained, and deployed in a matter of weeks or months (Langlotz et al., 2019). Unfortunately, this purported increase in efficiency comes with a price in terms of a significant increase in the opacity of the AI systems. Here follows a more detailed analysis of an application of DL to the prevention of breast cancer.

#### Deep Learning for Automated Breast Tissue Analysis

The starting point of the initiative is the medical notion that breast density is an important risk factor for breast cancer. Moreover, through their experience medical experts have found that areas of higher density can mask findings within mammograms (i.e., X-ray pictures of the breast used by doctors to look for early signs of breast cancer), which leads to a lower sensitivity, that is, fewer positive cases (i.e., patients with breast cancer) are correctly identified. Radiologists typically assess breast density by using the Breast Imaging Reporting and Data System (BI-RADS), which divides breast density into four categories: (a) almost entirely fatty; (b) scattered areas of fibroglandular density; (c) heterogeneously dense; and (d) extremely dense.

The assessment of BI-RADS breast density is performed by radiologists using digital images and, in such task, they exhibit intra-reader variability, i.e., measurement differences from the same radiologist, usually due to inherent difficulties with the digital image, and inter-reader variability, i.e., measurement differences among different readers, possibly because of a problematic digital image but more often due to differences in the readers’ skills. This can result in differences in estimated risk and, thus, differences in clinical care.

To tackle this issue, Matthews et al., (2020) proposed a DL system for breast density classification. The main difference between this DL-based experiment with the previous SVM-based proposal lies in the methodology followed by the AI system designers to enable it to perform classification of medical images.

In the SVM system there were explicitly quantified parameters given by the geometric model and by the thresholds of admissible distance from data points representing physiological corpora callosa. In the case of the DL system, the opacity of the “black box” is complete: the system is trained based on data pre-classified by experts without a specific and quantified indication on why the training data was classified that way. The neural networks in the DL system begin training with random mathematical parameters, which are then corrected and calibrated when the network classifies the training data incorrectly. When the classification errors fall below a pre-established threshold, the verification phase starts: the DL system is to classify a set of new data, the correct classification of which is known to the experts. If the system remains below an acceptable threshold of error rate, then it is considered ready for deployment: the AI system can classify new data, never classified before, neither by the system itself, nor by a human expert.

#### A Case Against Purely Data-driven Approaches in Medical AI Systems

To the entities involved in the planning and design of an SVM system listed before, new ones must now be added if DL technology is adopted:

- Those who choose the structural parameters of the neural network (also known as hyperparameters, they are not modified by the training, but they affect the performance of the network) and
- Those who provide the data with which the network is trained and then validated.

Accordingly, the potential causes for a false negative misdiagnosis increase. To the previously listed issues we must add the following:

- The medical image is classified as physiological by the network because of the setting of its parameters chosen by the network trainers;
- The medical image is classified as physiological by the network because it has been trained with data that did not feature the patterns characterizing that specific pathology.

These two cases of misdiagnosis shed light on two distinct although connected aspects of what working with ML systems implies. If we stick with the metaphor of the “black box”, these aspects can be thought of as dealing with what is fed to the box and what happens inside the box.

The most famous incident related to data given to an ML system did not occur in medicine, but on Twitter. “Tay” was the name of a chat-bot developed by Microsoft with the goal of interacting with Twitter users and learning from their tweets. After a mere couple of days Microsoft had to withdraw Tay because it was sending out tweets filled with racist, sexist, and antisemitic slurs. Mischievousness of Internet trolls aside, scholars consider this case a symptom of an intrinsic problem characterizing ML systems. In the context of software that “learns” from external data, the programmers have additional responsibilities on top of the already existing ones related to the encoding of knowledge into the software: they must go beyond the boundaries of their standard practice with traditional non-learning programs and be aware of and deal with all the possible results of the learning (Wolf et al., 2017).

To tackle the problems of understanding what happens inside the black box of an ML system, a new subfield of AI, called XAI (eXplainable Artificial Intelligence, Gunning et al., 2019), has recently emerged. The basic idea is to enhance an existing ML system so that its output is accompanied by an “explanation” of the results. Such explanation is for the humans using the ML system, and it is meant to provide information on how the system has reached the results in the output. This research field is still at an early stage because the XAI community has not yet agreed on a definition of what constitutes a proper explanation in this context. It is not even clear whether a unique definition will ever be possible, since there are several research efforts along different directions tackling many varied aspects of ML systems. For instance, in the context of image analysis, explanations can be based on the concept of “salience”: the ML system returns not only the classification of an image, but also an indication of the areas in the image that have contributed most, in terms of the weight of the factors involved, to that result. The strategy is to show the human expert a visualization in which the salient points that led to the result given by the system are highlighted. Other proposals for explanations include verbal reconstructions of the operations of the system and mathematical functions that are meant to model the relation between input and output values, among other techniques (Tjoa & Guan, 2020).

Whatever the nature and shape of the explanation, the goal of XAI is to make the “black box” of ML systems less “black” and more transparent, which is undoubtedly a praiseworthy effort. This, however, introduces a further type of problematic cases: to whom to attribute the responsibility for a misdiagnosis in which the attention of the clinician was focused on a particular area of the medical image indicated by the AI (or XAI) system, while in reality the truly critical area connected to the patient’s pathology was elsewhere and it was neglected by the clinician precisely because of these enhancements of the AI system that had been introduced to improve the use of AI in medicine? This problem is not new, and it is not limited to medical diagnosis: *automation bias* is an issue that has been discussed and tackled for decades in any context where humans must take decisions in a highly automatized environment (Skitka et al., 1999).

The principle of confidence may indicate the path towards reasonable solutions. XAI systems create indeed new opportunities for risk (e.g., misleading, erroneous explanations), or even harm in a full automation scenario. However, the XAI efforts towards the opening of black boxes, even if an exhaustive explanation may be out of reach, may ensure an increasingly higher level of transparency, which, in turn, could allow for an attitude of “alert confidence” by the doctors involved in the use of the AI systems. Concretely, this means that if evident indicators provided by the XAI make it possible to recognise that the suggested medical solution is not reliable, then the practitioner should reassess the case and independently take a responsible decision, either confirming the automated suggestion or taking a new course of action according to their individual clinical expertise and experience.

This scenario could reasonably meet the “meaningful human control” normative standard.

## Conclusions

A new, more conscientious way of using AI in medicine would rest on a compromise between machine automation and human responsibility that enables doctors to harness the technology without being overtaken by it. It is a very complex enterprise, because it is not just a technological issue, but a *sociotechnical* problem that touches on many and varied facets of society and hence requires a series of collective actions involving governments, markets, and communities (De Neufville & Baum, 2021).

Our reflection in this work has focused on the opportunity to hold the healthcare professional who makes use of AI systems responsible, without excluding the concurrent responsibility of other subjects involved in the design of those systems. We have seen that the number of associated stakeholders, especially when it comes to data-intensive technology, is significant. We acknowledge that attributing responsibilities by establishing a network of causal relationships would not be trivial in a field riddled by uncertainty like medicine. Indeed, if we shift from a purely ethical dimension to a legal context, the proof of causality between each individual conduct (especially for all subjects involved in the design of an AI system) and an adverse outcome would pose serious difficulties. However, this very complexity of the relationships between humans, organizations and technological artifacts confirms the usefulness of the principle of confidence, precisely because this normative instrument allows for the distribution of responsibility that is indeed shared in principle, but not yet in practice.

In this sense, our hope is that making all participants in the network aware of their responsibilities will help ensure a better distribution of duties among all the entities involved in the design, implementation, deployment, and use of AI systems in medicine, which should strike a balance between “*over*-responsibilization” and “*de*-responsibilization” for doctors. On the one hand, if the designers of an AI system perform and certify a quality control, then a doctor using that system should be in principle allowed to trust it without the need for continuous checks. On the other hand, if a wrong diagnosis depends on an error of the AI system, the concurrent responsibility of the doctor will depend on the possibility of recognizing that error and on their recklessness in acting. So, for example, if the AI system provides outputs that are significantly different from what the doctor has become accustomed to during an extended use of the system, then they would have the duty to verify the operation of the machine, possibly involving other people (e.g., the AI designers).

However, when ML systems are used, the opacity of the AI system prevents human users from grasping the automated decision-making process that led to a particular output. In such circumstances an approach based on confidence should be deemed imprudent, and it may be more appropriate to establish that the doctor must act (e.g., make a diagnosis) independently of the AI system, regardless of visible signs of malfunction.

In this regard, our point is not to take human medical doctors as an absolute “gold standard” to evaluate and compare the reliability of “black box” systems. While considering the relationship between *practitioners and AI systems* as analogous to the one between *practitioners and EBM* (medical guidelines or emerging research evidence), we could be tempted to claim that practitioners should comply with a sort of «epistemic obligation to align their medical verdicts with those of AI systems».^49^ Instead, full automation and “black box medicine” should be rejected on the grounds of the principle of responsibility which applies to medical doctors and the bioethical principle of autonomy which entails the patient’s rights to receive reasonable explanations and not to be treated unless they give their informed consent.

In conclusion, a reasonable regulatory model should allow for the combination of at least three requirements:

- (i) The right of patients to receive an explanation on the treatments they are receiving, which is a corollary of the fundamental bioethical principle of autonomy, so that they are not subjected to treatments against their will.
- (ii) The duty of doctors and all subjects involved in the design and operation of AI systems in medicine to protect the life and integrity of patients, a duty stemming from the principle of individual responsibility.
- (iii) The possibility for doctors to reasonably trust the tools at their disposal, unless otherwise prescribed, and to be able to notice clues of error of the system and consequently take a new course of action.

Considerations on technology, accuracy, and efficiency aside, it seems necessary to keep on focusing on ethical concerns about what opaque systems and full automation entail for patient-centered medicine. Responsibility must, in any case, be attributed to the humans who, in one way or another, participate in this very complex endeavor that is AI in medicine.

## Data Availability

Not applicable.

## Footnotes

1 This research endeavor, known as “machine ethics” (Floridi & Sanders, 2004), “artificial morality” (Allen et al., 2005), or “robot ethics” (Loh, 2019) is indeed aimed at conceiving and creating autonomous machines suitable to be treated as “moral entities”. The term “machine ethics” (Van Wynsberghe & Robbins, 2019) was first used in 1987 by Mitchell Waldrop in the AI Magazine article “A Question of Responsibility” (Waldrop, 1987). In 2005, the Association for the Advancement of Artificial Intelligence (AAAI) held a symposium on machine ethics which resulted in the edited volume *Machine Ethics*, edited by Susan Leigh and Michael Anderson in 2011 (Anderson & Anderson, 2011).

2 See for example Floridi & Sanders (2004), Allen et al. (2005). According to Van Wynsberghe & Robbins (2019, p. 721), the reasons often given for developing AMAs are: the prevention of harm, the necessity for public trust, the prevention of immoral use, such machines are better moral reasoners than humans, and building these machines would lead to a better understanding of human morality.

3 See for example Hallevy (2015) about the possibility of punishment of AI systems; see Mulligan (2018) for a similar stance.

4 The idea, which is still dominant within the Western tradition, is that “voluntary” misconduct is the essence of “fault” (Hegel 1821, §§ 117–118). Therefore, “no one should be punished unless he has clearly acted immorally, i.e., voluntarily harmed someone” (Hall 1963, p. 635–636).

5 Among them, Strawson expressed his basic argument as follows: “When one acts, one acts in the way one does because of the way one is. So, to be truly morally responsible for one’s actions, one would have to be truly responsible for the way one is: one would have to be *causa sui*, or the cause of oneself, at least in certain crucial mental respects. But nothing can be *causa sui* – nothing can be the ultimate cause of itself in any respect. So, nothing can be truly morally responsible” (Strawson, 1998). This argument recovers an idea expressed by Schopenhauer (1840, p. 84, 116): “the human being always does what he wills, and, indeed, he does it necessarily. However, this depends on the fact that he already *is* what he wills: for from that which he *is* everything that he ever does follows of necessity”. Therefore: “*Everything that occurs, from the greatest to the least, occurs necessarily*. (…) Our deeds are simply no first beginning, for in them nothing actually new attains existence: rather, *through that which we do, we simply experience what we are*”. The same idea is also found in Nietzsche (1878), §§ 18, 39, 99, 102, 106.

6 This position may underlie the recognition of “moral luck” (Nagel, 1979). In fact, “moral luck”, as it is broadly understood, occurs precisely “when an agent can be correctly treated as an object of moral judgment even though a significant aspect of what she is assessed for depends on factors beyond her control” (Nelkin, 2019). In this context, the factors beyond the agent’s control that nevertheless determine the agent’s responsibility are those biological and environmental factors that lead the agent to be *who they are* and therefore to assume certain behaviors (the so-called “nature” and “nurture”).

7 This conclusion poses the philosophical and practical question of the definition of this sphere of “normality” (Günther, 2006), “lack of constraint” (Ayer, 1972) or “responsible will” (Frankfurt, 1971), but it lies beyond the scope of this work. The question refers to the definition of the normative notion of “imputability”, *Zurechnungsfähigkeit*, as “personal *status*” about the “capability” of being the recipient of consequences, and it presupposes a “demonstrated ability to be accountable” (Donini, 2020, p. 1701).

8 As shown by the notes of Planck in *La conoscenza del mondo fisico* (1964, Bollati Boringhieri) quoted by Casertano (1975, p. 144 and following). This idea was already proposed by Feuerbach, who prescribed determinism in the juridical field and non-determinism in the moral field, and by Kelsen, in the theory of conditioned will (i.e., not free from conditioning and not suspended in an etiological vacuum) as an explanation of responsibility (Donini, 2020).

9 See footnote 7.

10 The reference to concurrent circumstances alludes to the criterion of the judgment of responsibility known in philosophy of law as *Zumutbarkeit* (Fornasari, 1990). The imputation of personal responsibility for one’s action requires evaluating whether the agent can be possibly excused for being unable to comply with their duty of conduct. According to that criterion, those who violate a rule of conduct under an exceptional “motivational pressure” determined by “exceptional” factual circumstances may be “excused” because those circumstances made them unable to do otherwise. They could not be “normally” motivated by the norm of conduct because of those “exceptional” circumstances.

11 In terms of “moral luck”. See footnote 6.

12 See also Tollon (2020).

13 Véliz (2021, Sect. 4 and following) analyzes meanings of “autonomy” (personal efficacy, psychological independence, or independence of mind, having a moral right to self-determination, authenticity, having a coherent self-image, being heroic, self-governance, and being responsive to reasons), some relevant to moral responsibility (e.g., self-governance and reasons-responsiveness), others not, but all inapplicable to AI systems.

14 A prejudice “inherited from the Christian tradition”, according to Ross (1970, p. 284–285). Instead, we can assume that everything that happens, happens out of necessity, and that the will is nothing more than the subjective perception of objective necessity (Schopenhauer, 1840).

15 “Rehabilitation and reintegration would therefore replace punishment as the focus of the criminal justice system” (Caruso, 2019, p. 61).

16 Schopenhauer (1840, p. 84) came to the same conclusion and showed that it was “the most abundant source of comfort and peace of mind”. For some overview on *liability* and *culpability* without *moral guilt*, from Nietzsche to 20^th^ Century criminal law, see Donini (2020). Instead, Mulligan (2018) argues for robots’ agency and therefore capacity to be blamed based on a “psychological instinct to blame” in humans.

17 See Donini (2020, p. 1719). For example, Italian positivism in criminal law referred to biological causes (Cesare Lombroso), psychological causes (Raffaele Garofalo), social causes (Enrico Ferri). Franz von Liszt, chief member of the so-called German “Moderne Schule”, in his famous Marburg Program (Liszt, 1883), classified delinquents into three categories: recoverable, irrecoverable, occasional.

18 Donini (2020), p. 1724–1725.

19 See Brown and Pratt (2000) for criticism.

20 Art. 3 of the European Convention of Human Rights states that no one shall be subjected to torture or to inhuman or degrading treatment or punishment. See Jehle et al. (2021), also for criticism about the current situation in Europe.

21 Available at https://digital-strategy.ec.europa.eu/en/library/proposal-regulation-laying-down-harmonised-rules-artificial-intelligence. It is important to clarify that it is a proposal, so it may take time before it enters into force in all 27 EU Member States as a Regulation. See Floridi (2021).

22 About the classification of medical devices as “high risk” artifacts pursuant to the AIA-21, see art. 6(1) and Annex II. See also the Explanatory Memorandum, Sect. 5.2.3, and the Purposes (30), (31) and (32).

23 Meaningful human control (MHC) is an expression introduced by Article 36, Killer Robots: UK Government Policy on Fully Autonomous Weapons, April 2013 p. 4: http://www.article36.org/wp-content/uploads/2013/04/Policy_Paper1.pdf. Article 36 is a British non-governmental organisation that aims at political and legal control to prevent harm from the use of lethal autonomous weapons.

24 GDPR-16, Art. 22.1: “The data subject shall have the right not to be subject to a decision based solely on automated processing, including profiling, which produces legal effects concerning him or her or similarly significantly affects him or her”.

25 See also Bjerring and Busch 2021.

26 Dealing with the application of this general prohibition to healthcare providers, some authors already argued that this legal tool is “adequate but not sufficient to address the legal, ethical and social challenges that Machine Learning technologies pose to patients’ rights and health care givers’ capacities”. It is unclear what this prohibition could mean in practical terms (De Miguel et al., 2020, p. 7).

27 Art. 22.3 establishes the “right to obtain human intervention on the part of the controller, to express his or her point of view and to contest the decision”.

28 Art. 14: “High-risk AI systems shall be designed and developed in such a way, including with appropriate human–machine interface tools, that they can be effectively overseen by natural persons during the period in which the AI system is in use. / Human oversight shall aim at preventing or minimising the risks to health, safety or fundamental rights that may emerge when a high-risk AI system is used in accordance with its intended purpose or under conditions of reasonably foreseeable misuse, in particular when such risks persist notwithstanding the application of other requirements set out in this Chapter”.

29 Art. 22, Duty of information: “Where the high-risk AI system presents a risk within the meaning of Article 65(1) and that risk is known to the provider of the system, that provider shall immediately inform the national competent authorities of the Member States in which it made the system available and, where applicable, the notified body that issued a certificate for the high-risk AI system, in particular of the non-compliance and of any corrective actions taken.”.

30 Art. 62: “Providers of high-risk AI systems placed on the Union market shall report any serious incident or any malfunctioning of those systems which constitutes a breach of obligations under Union law intended to protect fundamental rights to the market surveillance authorities of the Member States where that incident or breach occurred. / Such notification shall be made immediately after the provider has established a causal link between the AI system and the incident or malfunctioning or the reasonable likelihood of such a link, and, in any event, not later than 15 days after the providers becomes aware of the serious incident or of the malfunctioning.”.

31 For more references regarding legal perspectives and proposals based on the “meaningful human control” standard, see Braun et al., (2020, p. 6–7).

32 “How is it possible to train an artificial intelligence to be better than a human, if the data you give it is of the same low quality as produced by humans?” (Harvey, 2017). On the same argument, see also Cabitza et al. (2019) and Zaffalon et al. (2003).

33 See in this regard Art. 15(1–2-3), AIA-21: “High-risk AI systems shall be designed and developed in such a way that they achieve, in the light of their intended purpose, an appropriate level of accuracy, robustness and cybersecurity, and perform consistently in those respects throughout their lifecycle / The levels of accuracy and the relevant accuracy metrics of high-risk AI systems shall be declared in the accompanying instructions of use. (…) / High-risk AI systems shall be resilient as regards errors, faults or inconsistencies that may occur within the system or the environment in which the system operates, in particular due to their interaction with natural persons or other systems”.

34 See at this regard Art. 15(3–4), AIA-21: “The robustness of high-risk AI systems may be achieved through technical redundancy solutions, which may include backup or fail-safe plans. (…) / High-risk AI systems shall be resilient as regards attempts by unauthorised third parties to alter their use or performance by exploiting the system vulnerabilities. The technical solutions aimed at ensuring the cybersecurity of high-risk AI systems shall be appropriate to the relevant circumstances and the risks. The technical solutions to address AI specific vulnerabilities shall include, where appropriate, measures to prevent and control for attacks trying to manipulate the training dataset (‘data poisoning’), inputs designed to cause the model to make a mistake (‘adversarial examples’), or model flaws”.

35 See on this: European Commission, 2018; Ching et al., 2018; Grote & Berens, 2020.

36 See the interpretation of articles 13, 14, 22(1), 22(3) provided by the Article 29 Data Protection Working Party, “Guidelines on automated individual decision-making and profiling for the purposes of Regulation 2016/679”, EU Commission, 22 August 2018, p. 9, available at https://ec.europa.eu/info/law/law-topic/data-protection_en. See also Rubel et al., (2021, p. 87 and following), specifically on technical limitations associated to the three categories of explanations that the right to explanation could encompass: (1) ex ante system functional, (2) ex post system functional, (3) and ex post specific.

37 Art. 13, AIA-21: “High-risk AI systems shall be designed and developed in such a way to ensure that their operation is sufficiently transparent to enable users to interpret the system’s output and use it appropriately”.

38 See also Donovan (1956), 44: “There is no necessity to go to extremes in automation as applied to medicine. Medicine by its very nature is a combination of art and science”.

39 As Dr. Annalisa Manara recalls, “the patient did not meet the national criteria for coronavirus testing but because of his severe situation, we decided to do it anyway”: https://www.escardio.org/Education/COVID-19-and-Cardiology/diagnosing-the-first-covid-19-patient-in-italy-codogno.

40 David Silver interviewed by Lex Fridman, podcast #86 “David Silver: AlphaGo, AlphaZero, and Deep Reinforcement Learning”: https://www.youtube.com/watch?v=uPUEq8d73JI&t=5692s.

41 For example, exploring the standards currently set in comparative psychology (Halina 2021, p. 318).

42 A concept meant here as “an ability to interpret and reappropriate the fabric of socio-cultural expectations that can be (and regularly is) transformed through creative intervention” (Delacroix, 2021).

43 Halina (2021, p. 321, 323) concludes that, although AlphaGo lacks some of the virtues of animal “insight” (the “capacity to solve novel problems through a domain-general understanding of the world”), it exhibits the “capacity to transform a conceptual space in ways that do not appear available to human minds”, being able to produce “esults that are surprising, novel and valuable”. As we already observed, this also confirms that the answers to the more general question on whether AI systems has capacity for insight and generate creative or surprising outcomes depend on the paradigm of “creativity” that we have in mind (Halina, 2021, p. 326). Here, the author refers to the notion of “transformational creativity” and explicitly adopts Margaret Boden’s definition of “creative products” as “novel, surprising and valuable”. Compare further in the text Delacroix’s considerations about the required “surprise”.

44 Broadly speaking, EBM is a contemporary paradigm of medicine which combines three fundamental requirements: individual clinical expertise, scientific research, and patients’ autonomy (informed consent). According to its second pillar, medical decisions ought to be made based on the most up-to-date and reliable scientific evidence. Therefore, EBM provides doctors with best practices and medical guidelines. See, also for more references and current discussions, Sackett et al (1996), Rogers and Hutchison (2015), Szajewska (2018).

45 As London (2019, p. 18) observes, “Medicine is (…) a sphere where current theories of disease pathophysiology or drug mechanism are often of unknown or uncertain value”. Therefore, “the overreliance on theories that explain why something might be the case has sometimes made it more difficult to validate the empirical claims derived from such theories, with disastrous effects”. On controversial medical knowledge, its communication to nonexperts in legal, social, and clinical contexts, and the relationships between overreliance on science and social lack of trust, see also Perin, 2021.

46 Similar indications appear in art. 14, AIA-21: measures of human oversight shall enable humans “to decide, in any particular situation, not to use the high-risk AI system or otherwise disregard, override or reverse the output of the high-risk AI system”.

47 Accordingly, art. 29 of the AIA-21 established that: “e systems, pursuant to paragraphs 2 and 5”.

48 Consider that the AIA-21 established providers’ duties to notify adverse events (post-release).

49 Bjerring & Busch, 2021. See again on this, also for criticism, Braun et al., 2020; and Grote & Berens, 2020, p. 210, who arguably observe that “EBM is a movement within healthcare, whereas in machine learning the tech industry and leading computer science departments are among the major drivers. Suffice it to say at this point that the engagement of the industry entails ethical problems of its own”.
